# Depression Severity Is Different in Dysosmic Patients Who Have Experienced Traumatic Brain Injury Compared with Those Who Have Not

**DOI:** 10.3390/neurolint15020040

**Published:** 2023-05-12

**Authors:** Agnieszka Sabiniewicz, Kyri-Kristin Lindner, Antje Haehner, Thomas Hummel

**Affiliations:** 1 Smell & Taste Clinic, Department of Otorhinolaryngology, Faculty of Medicine Carl Gustav Carus, Technische Universität Dresden, 01307 Dresden, Germany; 2 Institute of Psychology, University of Wrocław, 50-527 Wrocław, Poland

**Keywords:** traumatic brain injury, olfaction disorders, quality of life, depression

## Abstract

Traumatic brain injury (TBI) in humans can result in olfactory, cognitive, and affective changes. Surprisingly, research on the consequences of TBI often did not control for olfactory function in the investigated groups. Consequently, the affective or cognitive differences might be misleading as related rather to different olfactory performance than to a TBI experience. Hence, our study aimed to investigate whether TBI occurrence would lead to altered affective and cognitive functioning in two groups of dysosmic patients, one with TBI experience and one without. In total, 51 patients with TBI experience and 50 controls with varied causes of olfactory loss were thoroughly examined in terms of olfactory, cognitive, and affective performance. Student *t*-tests demonstrated that the only significant difference between the groups appeared in the depression severity, with TBI patients being more depressed (t = 2.3, *p* = 0.011, Cohen’s d = −0.47). Regression analyses further showed that TBI experience was significantly associated with depression severity (R2 = 0.05, F [1, 96] = 5.5, *p* = 0.021, beta = 1.4). In conclusion, the present study showed that TBI experience is linked to depression, which is more pronounced compared to individuals with olfactory loss without TBI.

## 1. Introduction

Traumatic brain injury (TBI) in humans can result in a wide spectrum of consequences. Blunt injury to the head mainly affects the frontal and temporal regions and results in a number of cognitive, physical or psychosocial deficits [1,2]. Cognitive changes in patients with TBI experience have been the focus of attention [3,4,5]. Slowed thinking or difficulties in concentration and memory appear to be commonly mentioned in the first years after the trauma [1]. However, possible cognitive consequences can be reflected in a variety of other complications, such as problems with attention [6], executive functions [7], language functions [8] and visuospatial processing [9].

Apart from cognitive consequences, TBI may bring various psychiatric problems [10], with depression being one of the most common disorders [11]. In TBI patients, the risk of developing depression is higher compared with the general population, even several years after the injury [11,12]. Specifically, approximately 33–42% [13,14] of all TBI cases will result in depression within the first year, and depression emerges in more than half of the patients within seven years of the trauma [15,16].

Lastly, TBI, in terms of physical changes, is one of the leading causes of smell disorders [17,18,19,20], the frequency of which varies from 4% to 69% [21]. The degree of a disturbed sense of smell was found to range from slightly decreased to completely anosmic [22]. Even though recent evidence [20] suggests some recovery of olfactory function within the first six months following a TBI, the broad extent of olfactory disruption after TBI calls attention.

Considering the large number of post-TBI olfactory alterations, a closer look should be taken at their significance. Olfactory dysfunction has a strong impact on the life of patients and may give rise to a plethora of problems, including cognitive [23] or affective changes [24]. Regarding the latter, as many as 17% to 30% of patients with olfactory disorders state symptoms of depression [25,26]. Furthermore, change in olfactory functions has been shown to be linked to cognitive changes [23,27,28]. The exact reasons behind this process are still unknown, but presumably, brain degeneration appears first in olfactory-related circuits [29,30,31].

Surprisingly, research on the consequences of TBI often did not control for olfactory function, while neglecting this aspect results in matching normosmic individuals (having a normal sense of smell) to dysosmic patients (having a distorted sense of smell). Consequently, the affective or cognitive differences might be, in this case, misleading as related rather to different olfactory performance than to a TBI experience. Thus, our study aimed to investigate whether TBI occurrence would be associated with altered affective and cognitive functioning in two groups of dysosmic patients, one with TBI experience and one without. It was hypothesized that the TBI group would have lower performance in cognitive tests, indicating lower cognitive functions, and higher performance in affective tests, indicating higher depression severity.

## 2. Materials and Methods

### 2.1. Participants

Detailed characteristics of participants called further patients are presented in Table 1.

All patients visited the Smell and Taste Clinic of the Department of Otolaryngology (ORL) because of olfactory loss. Consecutive patients were recruited in the above-mentioned Clinic from August 2016 to December 2017 by being directly approached by the researcher and asked to participate. Since the recruitment was conducted among patients visiting the Clinic, an oral invitation given by the researcher, together with a short description of the study, was given to initiate the enrollment procedure. Patients of the TBI group visited the clinic specifically because of posttraumatic olfactory disorders. Patients from the control group had varied causes of olfactory loss, including chronic rhinosinusitis, postinfectious olfactory loss, Parkinson’s disease, or congenital anosmia.

The diagnosis of TBI was made on the basis of a thorough, structured history involving the exploration of patients’ clinical records, including MRI of the head. All patients received a detailed otorhinolaryngological examination, including nasal endoscopy. Eventually, all patients filled in questionnaires concerning their health.

Data were collected at the Smell & Taste Clinic of the Department of Otorhinolaryngology of the TU Dresden from August 2016 to December 2017.

### 2.2. Inclusion and Exclusion Criteria

Inclusion criterion for all patients was olfactory loss and age ≥ 18 years. Exclusion criterion was major cognitive dysfunction.

### 2.3. TBI Experience

The duration from the occurrence of TBI until the date of the test varied from less than one month up to 23 years with this interval being less than one year in 51% of cases. Additionally, 63% of the patients from the TBI group experienced a facial fracture, and 37% did not. All patients indicated that they had normal olfactory function prior to TBI that had caused olfactory loss.

### 2.4. Olfactory Testing

The detailed results of olfactory, cognitive and affective tests are presented in Table 2.

Olfactory testing was performed by means of “Sniffin’ Sticks”, a well-characterized tool to measure olfactory performance [32,33]. Here, it consisted of tests for odor threshold (rose-like odor, phenylethylalcohol; PEA), odor discrimination, and odor identification. Results of the three subtests were presented both separately for threshold (T), with a range between 1 and 16, discrimination (D), with a range between 0 and 16 and identification (I) score, with a range between 0 and 16, and as a sum of the results (TDI), with the final score ranging between 1 and 48 points [33]. If the TDI score was 31 or higher, the patient was regarded as normosmic; with a score lower than 16.5, the patient was considered anosmic. While in the TBI group decrease in olfaction was caused by TBI occurrence, in the control group, the reason was the presence of chronic rhinosinusitis (73%), Parkinson’s (4%) and Alzheimer’s (17%) disease running in the family (see Table 1).

Furthermore, the degree of parosmia (distorted odor perceptions in the presence of an odor source) and phantosmias (odor percepts in the absence of an odor) was assessed based on the patients’ structured medical history. Minimum score was 0 and maximum score was 3 points [34] (for parosmia: M = 0.61, SD = 1.0, for phantosmias: M = 0.21, SD = 0.6).

### 2.5. Questionnaires and Neuropsychological Tests

#### 2.5.1. Cognitive Testing

Wisconsin Card Sorting Test (WCST) [35] was employed to measure executive functioning. WCST is one of the most extensively used tests as it is reportedly sensitive to brain dysfunction affecting the frontal lobes [36]. In its conventional form, patients are asked to sort a series of cards by dividing them into four piles. The cards vary in terms of three attributes: the number, color and shape of their elements. The categorizing starts with color, followed by shape and number, and then the procedure is repeated in the same order. After each response, the feedback is given to the participant (‘correct’ vs. ‘incorrect’) that serves to set up the correct sorting rule. After ten correct responses in a row, the sorting rule changes without warning (‘completing a category’) and the participant must ‘start again’ to find out the new sorting rule for the given category [37]. The task is finished after two decks of 64 cards are sorted or after six full categories are achieved.

Controlled Oral Word Association (COWA) [38] was used to assess verbal fluency. The test evaluates how many words the participant is able to produce spontaneously within a limited amount of time, beginning with a given letter of the alphabet [39]. Neither proper nouns (e.g., Carl, California) nor saying the same word using a different ending (e.g., cancel, cancelled) should be included. The test is composed of three trails, each employing a different letter (e.g., FAS, CFL). Participants are allowed 60 s for each trial. The COWA is a sensitive measure of brain deterioration and the comprehensive assessment of neuropsychological functioning [39].

The Trail Making Test, part A (TMT) [40], provided information on executive functions. TMT is a neuropsychological test that involves visual scanning and working memory. It is commonly employed as a screening instrument for detecting neurological disease and neuropsychological deterioration [41]. A participant is asked to connect 25 consecutive circles in numerical sequence beginning with the number “1”. The TMT is scored by the time one takes to complete the test. In the case of TMT A, the average time to complete this part is 79 s.

d2 Test measured selective attention and concentration [42] by assessing the participant’s ability to selectively, quickly and accurately focus on certain relevant aspects in a task while ignoring the irrelevant ones [43]. More precisely, the task is to cancel out all target characters (a “d” with two dashes placed on top [d’’] and/or at the bottom [d,,]), among non-target characters (a “b” and “p” character with any number of dashes), in 14 successive timed trials [43].

#### 2.5.2. Affective Testing

**Figure 1:**
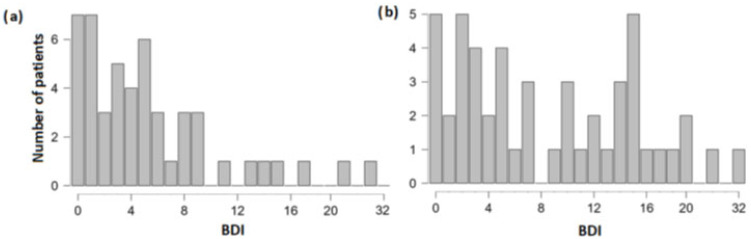
Depression severity in (**a**) control patients; and (**b**) TBI patients. Note different Y-axis sizes.

Beck-Depression Inventory (BDI) [44] was used to gauge the severity of depression. This self-reported instrument has scores that can range from 0 (symptom absent)–3 (severe symptoms) across 21 items, leading to the total score of 0–63. Higher scores indicate greater severity of depression (Figure 1). More specifically, affective, cognitive, somatic and vegetative symptoms are assessed in this inventory, reflecting the DSM-IV criteria for major depression [45].

Data are available on a reasonable request by emailing the corresponding author.

### 2.6. Ethical Issues

The study was performed according to the principles of the Declaration of Helsinki on biomedical research involving human subjects. It was approved by the Ethics Committee at the Medical Faculty of the TU Dresden (EK number 371082016, 1 September 2016). All patients provided written informed consent.

### 2.7. Data Analysis

We firstly aimed to investigate possible differences in (a) olfactory (separately for T, D, I and TDI), (b) cognitive (WCST, COWA, TMT and d2 test) and (c) depression (BDI) scores between the groups of patients with and without TBI experience via one-way unpaired *t*-test. Below, we present specific categories taken into consideration from each cognitive test.

In WCST, two categories were included in the analyses: perseverative errors (PE), and non-perseverative errors (NPE).

In COWA, the total number of words produced by patients was analyzed (TW).

In d2 Test, four categories were included in the analyses: a total number of characters processed (TN), errors of omission (O), errors of commission (C) and concentration performance (CP).

In TMT test, the average score (TMT A) of the four trials made by patients in part A was included in the analyses.

These analyses were conducted both in classic and Bayesian ways [46,47,48] to understand better the nature of the differences or the lack of differences between the groups. The Bayes Factor (B) is a method that weighs evidence and shows which out of two hypotheses: alternative hypothesis (H1) or null hypothesis (H0) is better supported. Adopting the BF in statistical inference, it can be shown whether data provided stronger support for the null hypothesis, the alternative hypothesis or whether it is inconclusive, and more data needs to be collected to provide more decisive evidence [49]. Furthermore, Bayesian statistics are resistant to multiple comparisons.

Secondly, to further examine whether two groups of dysosmic patients, one with TBI experience and one without, would differ in terms of cognitive (WCST NPE) and affective (BDI) performance, we ran classic linear regression analysis. More specifically, two separate models with two different dependent variables were conducted. In the first model, affective performance (BD) was inserted as a dependent variable and in the second model, cognitive performance (WCST NPE) was inserted as such. In both models, the independent variable was the classification of the TBI vs. control group.

Additionally, to investigate whether the duration since TBI occurrence (in months) correlated with depression severity, Pearson’s correlation was run between these variables.

In all the tests, raw outcomes were analyzed.

Data are presented as mean values (±standard deviations). Statistical analyses were performed using JASP v. 0.16.2 with a level of significance set to α = 0.05 (www.jasp-stats.org accessed on 1 August 2022).

## 3. Results

### 3.1. Differences in Olfactory, Cognitive and Depression Scores between the Group of Patients with and without TBI Experience

**Figure 2:**
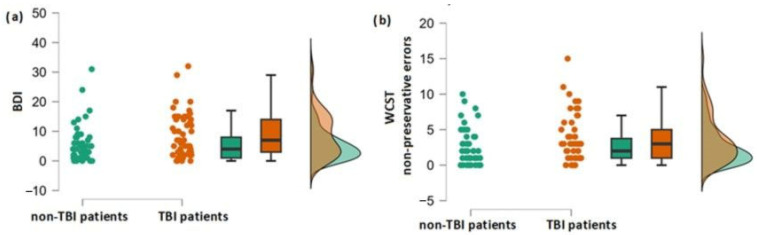
(**a**) Differences in depression severity between the group with and without TBI experience; (**b**) Differences in the number of non-preservative errors in WCST test between the group with and without TBI experience.

Classical *t*-test indicated that, in the case of the majority of the analyzed olfactory, cognitive and affective aspects, the two groups did not differ (Table 3). Only in case of WCST NPE (t = 1.9, *p* = 0.029, Cohen’s d = −0.38) and BDI (t = 2.3, *p* = 0.011, Cohen’s d = −0.47) the TBI group scored higher compared to the control group (Figure 2a,b).

Bayesian *t*-test demonstrated that the two groups were indistinguishable from each other in terms of olfactory performance (for T: B01 = 8, for D: B01 = 4, for I: B01 = 10.2, for TDI: B01 = 7.7) and several components of cognitive performance (for d2 CP: B01 = 6.1, for WCST PE: B01 = 5.7, for COWA TW: B01 = 11.5). The only difference between the two groups occurred in the case of the BDI score, thereby confirming the results of the classic *t*-test (B10 = 4.6).

### 3.2. Association between TBI and Cognitive and Affective Performance

The regression model conducted for BDI indicated that experiencing TBI significantly influenced depression severity (R2 = 0.05, F[1, 96] = 5.5, *p* = 0.021, beta = 1.4). In the case of WCST NPE, the model was significant on a trend level only (R2 = 0.04, F[1, 98] = 3.7, *p* = 0.058, beta = 0.6).

### 3.3. Correlation between the Duration since TBI Occurrence and Depression Severity

Correlation analyses showed that the duration since TBI occurrence was unrelated to depression severity (r = −0.05, *p* = 0.594).

## 4. Discussion

The present study aimed to investigate whether two groups similar in terms of impaired olfactory performance and different concerning TBI experience would vary in cognitive performance and severity of depression. Olfactory function was measured by means of the extended Sniffin’ Sticks test battery [32], while the different aspects of cognitive performance were measured by WCTS [35], COWA [38], TMT [40] and d2 Test [42]. Additionally, BDI [44] was used to determine the severity of depression. Interestingly, while the two groups tended to differ regarding non-preservative errors (WCST NPE), the only significant difference appeared in the depression severity (BDI), with TBI patients being more depressed. The same pattern was visible in regression models, with TBI experience being significantly related to depression severity while cognitive abilities (WCST NPE) were influenced only on a trend level.

Beck-Depression Inventory (BDI) has been successfully employed among TBI patients in a basic diagnosis of depression [50] and to further determine whether depressive symptoms warrant assessment [51]. Parallelly, the usefulness of BDI has also been demonstrated in healthy people [52]. In the present study, TBI patients reported higher depression levels compared to the control group, which is in line with previous studies underlining the connection between TBI occurrence and depression manifestation [14,53,54].

The development of depression following a head injury is multifaceted. Outcome studies have indicated an interplay of several factors, such as a reduced capacity for functional independence, study, employment, leisure activities and personal and social relationships [55,56,57,58,59]. Ahmed and colleagues [60] reported that only approximately 25% of TBI patients achieve long-term functional independence. Moreover, persistent pain restricting activities may lead to a sense of victimization as the result of the head injury and, in consequence, to the sense of loss of control of one’s life [60]. This response appears particularly in the context of a prior history of physical, sexual, or emotional abuse or a combination of the three.

Considering neuroanatomical changes related to post-TBI depression, studies in this field showed damage to the central nervous system, especially in the olfactory bulb and frontal lobe [61,62,63,64]. Since these areas are particularly involved in emotion regulation, their damage may explain the rise in depression symptoms. Particularly interesting in the context of our research is a study by Han and colleagues [18], who examined the structural brain changes in patients with both TBI and olfactory loss when compared to hyposmic patients with TBI and healthy controls. Patients with anosmia had more frequent lesions in the olfactory bulb, orbitofrontal cortex, and the temporal lobe pole when compared with patients with hyposmia, with grey matter density reduction in several secondary olfactory eloquent regions. These changes have been found related to depression symptoms [65,66].

All the studies mentioned above underline the occurrence of post-TBI depression. Here, we are first to demonstrate that the depression severity was specifically associated with TBI experience even when compared to dysosmic patients, i.e., the ones who experienced similar olfactory impairment. In fact, the Bayesian factor demonstrated that the olfactory performance in all analyzed functions was indistinguishable in both groups. Although olfactory dysfunction is related to depression [24,53,67,68], the present findings emphasize the important role of TBI experience in causing depression.

A vast number of studies reported cognitive changes following TBI occurrence. Among all, impaired executive functions, attention, trouble shifting sets, deficits in verbal fluency or problems with working memory have been mentioned repeatedly [69,70,71], with alterations in the white matter given as a plausible explanation [72]. The present study that focused rigorously on a number of cognitive functions, such as executive functioning, verbal fluency, visual scanning, working memory, selective attention and concentration, measured with objectively validated methods, demonstrated only a trend effect regarding a higher number of non-preservative errors made in WCST test by TBI patients, and the link between TBI experience and the number of these errors. Since non-preservative errors, defined as all the random errors in the test, were reported to underline deficits in patients with prefrontal lesions [73], the present results fit into previous notions on the connection between TBI and cognitive impairment.

Still, these results need to be put into perspective. The question of why only one cognitive aspect appeared among all the investigated effects and solely on a trend level requires a more profound discussion. One plausible explanation is that both investigated groups did not differ in terms of olfactory performance. Olfaction is well-established to be a marker of cognitive performance [23] and to predict cognitive decline when impaired [74]. Wang and colleagues [75] demonstrated that olfactory dysfunction was already present with the subjectively reported cognitive decline, which suggests that the connection between these two is present on many levels. In contrast to other studies that did not control for olfactory performance or compare normosmic/hyposmic to anosmic people’s performance [76] and demonstrate a number of cognitive differences between those who did and did not experience TBI, here we compared two dysosmic groups and found that TBI occurrence had only a slight impact on cognitive outcome. Hence, we conclude that the consideration of olfactory function is crucial for the full appreciation of TBI-related cognitive consequences.

### 4.1. Limitations

Firstly, more accurate measures to assess the severity of TBI and brain damage, such as the Glasgow Coma Scale [77], should be included in future studies. Here, we cannot exclude other factors that could potentially cause the above effect. Several factors, such as posttraumatic amnesia duration or higher pre-morbid intelligence, have been demonstrated to play a role in cognitive performance [78]. Since we did not collect such information, future studies should further examine this issue. Likewise, confounders, such as intracranial injury location and severity, frontal/nasal bone/olfactory apparatus injury and severity, baseline history of depression or mental health disorders, etc., were not controlled for, and hence we cannot exclude their impact on the obtained results. Secondly, the follow-up after TBI experience varied from less than one month up to 23 years, which is a highly different experience. However, on the other hand, the long duration of the posttraumatic olfactory loss indicates that, in the present study, signs of depression are unlikely to be a consequence of short-term changes. Eventually, direct comparison between patients with posttraumatic anosmia and those with congenital anosmia is a limitation in itself.

### 4.2. Conclusions

Overall, the results of the present study show that in both groups, impaired in terms of olfaction, TBI resulted in higher depression severity, while no significant difference was found in terms of cognitive abilities. Hence, the first result is in line with previous studies that did not control for olfactory performance, while the second result is in contrast to the majority of the studies showing that TBI patients exhibit lower cognitive scores than patients without TBI experience. We assume that the discrepancy was associated with the presence of olfactory dysfunction in both groups. We conclude that olfactory performance is a measure to be taken into account in future studies on TBI. Practical implications are that TBI patients are often depressed and that this should be actively approached during the interview with the patients.

## Figures and Tables

**Table 1:** Descriptive statistics of patients.

	Women	Men	Total
Number of patients	38	63	101
Age (M *±* SD) in years	51.7 ± 15.3	52.6 ± 4.8	52.3 ± 14.9
	Group of TBI patients (*n* = 51)		Control group (*n* = 50)
Men %	65%		60%
Age (M ± SD)	51.9 ± 14.9		52.6 ± 15.1
Anosmic patients %	51%		44%
Cause of olfactory loss			
Traumatic brain injury	*N* = 51		-
Chronic rhinosinusitis	-		*N* = 11
Congenital anosmia	-		*N* = 3
Postinfectious olfactory loss	-		*N* = 13
Idiopathic olfactory dysfunction	-		*N* = 21
Parkinson disease	-		*N* = 2
Type of TBI	unspecific (33%); occipital (26%); frontal (18%); polytrauma (2%); no information available (22%)		-
Cause of TBI	accident (29%); fall (24%); fight (2%); other (12%); no information available (33%)		-

**Table 2:** Detailed results of the olfactory, cognitive and affective tests. T represents the olfactory threshold, D discrimination, I identification and TDI stands for the overall olfactory score. d2II TN represents the total number of items processed, d2II O errors of omission, d2II errors of commission, d2II CP stands for concentration performance. WCST PE identifies the number of preservative errors, and WCST NPE stands for the number of non-preservative errors. COWA T identifies the total number of words produced by the participant. TMT A indicates the average score of the four trials made by the participant in part A. BDI indicates Beck-Depression Inventory score.

Test Name	TBI Patients		Control Patients	
	M	SD	M	SD
Olfactory tests				
T	2.6	2.6	3	2.9
D	8	2.7	7.9	3.6
I	6	3	7	4
TDI	16.7	7.1	17.9	8.3
Cognitive tests				
d2II TN	148.6	29	144.1	42
d2II O	22.2	20.7	17.7	19.8
d2II C	5.5	15.2	2.8	3
d2II CP	120.8	39.2	123.6	129.4
WCST PE	7.3	3.2	7.5	2.3
WCST NPE	3.6	3.4	2.5	2.5
COWA T	60.8	15.2	66.4	15.1
TMT A	35.6	20.6	32.4	12.7
Affective test				
BDI	8.9	7.5	5.6	6.2

**Table 3:** *t*-test results for olfactory, cognitive and affective measures between the TBI and control groups.

Outcome	Cohen’s d	t	*p*	df
BDI	−0.47	2.3 *	0.011	96
COWA TW	0.37	1.8	0.958	89
WCST PE	0.05	0.3	0.599	98
WCST NPE	0.38	1.9	0.029	98
TMT A	−0.189	1	0.173	99
D2 TN	−0.12	0.6	0.268	99
D2 O	−0.22	1.1	0.132	99
D2 C	−0.24	1.2	0.112	99
D2 CP	0.07	0.4	0.639	99
TDI	0.15	0.8	0.421	99
T	0.16	0.9	0.828	99
D	−0.04	0.2	0.19	99
I	0.26	1.3	0.453	99

* *p* < 0.05.

## Data Availability

The datasets analyzed during the current study are not publicly available due to the privacy of the participants but are available from the corresponding author at reasonable request.
